# Dental Resources

**Published:** 1888-05

**Authors:** S. B. Palmer


### DENTAL RESOURCES.

BY S. B. PALMER, M. D. S.

READ BEFORE A UNION MEETING OF THE SIXTH, SEVENTH AND EIGHTH DISTRICT DENTAL SOCIETIES, AT BUFFALO, N. Y.

When a young man decides to enter the dental profession, the usual method is to spend some time in a dental office and conclude his studies at a dental college. A glance at the college notices in dental journals will show that there is no lack in numbers or grades of such institutions, with locations convenient for students in almost every section of the country.

Again, as you turn the pages of the voluminous catalogues furnished by dealers in dental supplies, it would seem that every article or appliance needed was therein described or illustrated. Enter the dental depots ând there may be found a complete outfit for all that is usually required in the office. Leaving college the student enters the higher school of practice, with possibilities as much above college training as the latter is above an office term. Up to this time, the way has been mapped out, and little more than attention to study is required to pass a creditable examination. The school of practice develops the capabilities of the student for his life work. It announces no special course of study, fixes no time for graduation. The studies are progressive, and all previous instructions are looked upon as merely preparatory, and things of the past. Dental periodicals take the place of text books, clinical instructions are received from practice, and society meetings more than fill the professors' chairs with practical and scientific lectures. The rapid growth of dentistry is the outcome of its many resources. When it was known that the new art was more lucrative than ordinary occupations, there was no lack of willing hands to engage in this new industry. From the barber to the surgeon, the jeweler to the sculptor, there came more or less skilled laborers, bringing with them the tools, appliances and materials used in their respective occupations. Wise legislation has restricted this influx of labor, but the contribution of materials remains and is a great help in the laboratory and operating room to-day. No one who is legally qualified to open an office in this State need feel discouraged, however limited in means or inexperienced in practice. If fortunate enough to possess a degree, the public will receive it for all it is worth, and to the holdor it is worth more than it cost.

Without this valuable recommendation, the way to public confidence is harder, and more private study will be required to make up the deficiency. Success, however, will depend upon merit, which, like blood, will tell. Education and opportunities may do much in establishing and maintaining a practice, yet a born dentist will grow to the full stature of a man. Retarding circumstances may hinder in early life, but once established the school is open alike to all, and by extra exertion one may make up time and come to the front. While the candidate for political office must answer for the sins of his parents as well as his own, the public generally judges of a dentist by what he appears to be, what he does, and by his standing with his professional brethren. Although modern practice requires a much higher grade of operations than in former years, the facilities for obtaining education, as well as convenient appliances, render the study of dentistry easy compared with the writer's early experience.

Many years since, a farmer boy living three miles from the nearest village and sixteen miles from the then village of Syracuse, was in need of a superior denture of nine teeth. Without ever having seen a dental plate or the inside of a dental office, without personally knowing any dentist except the one who, in modern language, was "on the road," and who did the extracting, this boy conceived the idea of doing the work for himself, or taking up dentistry without a master. Here commenced the study of resources. The first step was to see a plate. Through the kindness of a lady the article was examined, and a resolution formed to make the trial. It is needless to say that lack of funds stimulated action, and verified the adage that "necessity is the mother of invention." Upon inquiry, dental supplies were found at a drug store in Syracuse; also a book, "The Human Teeth, by Paul B. Goddard," published in 1884; price $5. The book was purchased. The investment seemed large, but tolerably safe. That old book is number one of, and a treasure in a professional library to-day. Though by no means the best work of the time, it was the only available one, and it served its purpose nobly. The cuts were excellent and the illustrations well defined. Study was limited to the one object in view, without the remotest idea of any practice of dentistry beyond that.

As the mind developed, machinery and appliances were improvised and held ready for use. An alcohol blowpipe was made, and experiments tried in hard soldering. A spinning wheel was converted into a grinding lathe, impressions, casts and dies were produced as directed in the book, and a silver coin reduced to a plate upon the anvil and by grinding. The plate was struck up, clasps soldered, tried in the mouth and pronounced a good fit. By the aid of the druggist's clerk and the articulating models the teeth were selected, and in time the case was finished at two o'clock in the morning, some six or seven months from the commencement. The size and style of the plate was exactly on a line with the engraving in the instructions, the fit and finish was as good as would be produced to-day. The work was mostly done at odd hours, and as fast as the mind could be educated to do it properly.

Following this first operation a few plates were made for friends. Excavators and pluggers were fashioned and teeth filled for members of the family, till the study of dentistry was finally decided upon. A course of office instruction followed, and in time practice was commenced, all of which was comparatively easy after the study mentioned above, and thus I became a dental practitioner.

With a will the way is now open to almost every undertaking. In dentistry, societies and dental journals are constant helps. No one need be behind the times in practice; none can be up in practice without such helps. There are too many dental hermits, men who confine themselves to their office demands and have little association with others outside. This habit has a tendency to narrow one's usefulness and circumscribe his practice. A few years devoted to extracting teeth and making plates would disqualify an operator for successfully treating roots and inserting crowns.

In early times, the northern portion of Onondaga County grew timber suited to the manufacturing of salt barrels, for which there was great demand. Almost every farm had its cooper shop. Competition was sharp and prices low. The quality of work turned out was as near the inspector's cull line as possible. This kind of cooperage was soon learned and made profitable, but when a regular mechanical workman went into this new specialty his education for good work was against him. He had not the sleight of hand to make the number necessary for competition. On the other extreme, when the timber was used up and the rapid workman undertook tight work, his failure was in quality instead of quantity.

To elevate dentistry, one must understand the ways and means to do it. It is very important to understand how little we know. This can be done by comparison, and gatherings like this afford the best opportunity for such knowledge. It may be regarded as a fact that no dentist can be in the front ranks who neglects to attend societies or to take the journals.

Perhaps the next most important help to dentistry is the outcome of dental appliances, in connection with dental depots. Dentistry made the demand, moneyed manufacturers experimented and furnished the supply. Notwithstanding centralization and combinations are detrimental to the consumer, this does not alter the fact above stated. There are two sides to this question of combination. Every trade strives to protect its own interest. When dentistry looks through its spy-glass, it sees a large monopoly constantly collecting tribute from the profession. When patients turn their glass upon dentistry, they see a combined sentiment that operations are worth all that can be obtained for them, and bills rendered for all they are worth. If dentistry will turn the glass and look in the other end, it will see that another man's bull has been gored.

Calling upon dentists of known ability is another means of information. Some good points are sure to be gained. Don't form hasty opinions of new methods of operations because of criticisms. Nearly every advance of any value had to undergo this ordeal. "Survival of the Fittest" is the foundation of practice. No one method or material is best for all, as may be seen in the manipulation of gold. In proper hands good work is done with each variety. The requirements are, that the gold must be held in absolute contact with the walls of the cavity; a thorough knowledge of the working of various preparations gives an operator the advantage of adaptation to circumstances. Again, there are extremists, or specialists, in operative dentistry, both working for the same end —tooth preservation. It delighted the late Dr. Webb to give the profession the highest type of instructions in contouring and finishing fillings of cohesive gold. His work upon this subject must stimulate every operator to do better and aim higher in this direction. At the other extreme, Dr. Flagg's work on "Plastics and Plastic Fillings" will be found equally important, as it teaches the special conditions of teeth and their relations to other filling materials for preservation far beyond the possibilities of gold to accomplish. The prudent operator informs himself of both methods, and thereby is able to adapt materials to circumstances.

It is a privilege, and often a convenience, to obtain articles outside of the trade. This can be done by means of circulars and catalogues. Every trade or industry has its price list, and generally an illustrated catalogue. It has been my pleasure to read the "Scientific American" for thirty years. When the address of any firm or trade is wanted, a letter addressed to that paper has given the information, and thus the way is open for correspondence. As opportunity occurs, it is well to obtain catalogues of various trades. None are more beneficial to a dentist than the jeweler's. In some respects it is better for many articles used in the laboratory than a dental catalogue.

A perusal of the illustrated catalogue published by F. W. Gesswein, No. 39 John Street, New York, will show polishing wheels, brushes and polishing powders in great variety at figures as low as the trade will warrant, and with no combination to secure exorbitant rates.

A large druggist's catalogue, published by Whitall, Tatum & Co., 410 Race Street, Philadelphia, Pa., will give cuts of syringes, brushes, glass bottles, boxes, labels and every article in that line.

James W. Queen & Co., 924 Chestnut Street, Philadelphia, publish sixteen catalogues, some of which contain important information for a dentist. Send for a condensed catalogue, which describes the contents of the others.

I wish to give Dr. H. C. Merriam, of Salem, Mass., credit for many valuable suggestions given in a clinic at the Congress.

For dry grinding of almost any material in the laboratory, use wheels of emery and corundum. Send for circular to Vitrified Wheel Co., Westfield, Mass., or to the Waltham Emery Wheel Co., as advertised in the INDEPENDENT PRACTITIONER. Soldering blocks made of boiler covering, asbestos and magnesia, light, clean, non-conducting and very cheap, can be obtained at boiler shops.

Small polishing wheels and points for cleansing teeth may be made from Faber's ink erasers. Twist drills may be made from piano wire without drawing the temper. They cut rapidly, and will drill on a curve without breaking. The manufacture of them has been placed with Philip Schmidt, 218 West Twentieth Street, New York, who is well known as a first-class repairer of dental instruments. In polishing with felt, use two wheels upon the mandrel. The space between retains the powder, and the piece, if thin, may be polished on the edge and sides at the same time.

Leaving this clinic I will add that waste amalgam may be converted into a new article, even better than the average of the original, with very little trouble or expense. Place the waste in a crucible without flux, heat to redness for ten minutes, weigh the ingot thus obtained, and to each ounce add five pennyweights of pure silver, two pennyweights of tin, and eight grains of gold. Melt under borax. No more heat is required than to make the mass fluid. Stir with an iron wire, and cast the ingot in convenient shape for filling. A good alloy will be the result.

Very few seem to appreciate the advantage of stoning such burs as are often needed for special use. Procure the knife edged Arkansas stone slips, place the bur in the mandrel of an old hand piece, or a socket made for holding burs, wet the stone with glycerine and water, or with light oil, and use the stone as you would a file till the edges are sharp. It is well to work with a glass. Care is necessary or the stone will become nicked. Keeping the stone in order is the most particular part of the operation. This is done with fine emery paper. Cut a sheet of paper into strips about two inches wide, the shortest way of the sheet. Make the strips into a pad upon a piece of wood the same width and about two inches longer. Secure the first paper to the wood and the ends of the pad with glue or mucilage. In using, the upper leaf may be torn off when the grit has been used up. To prevent nicking, the stone should be drawn parallel with the pad. The emery cuts rapidly, and a sharp, thin edge can be produced in a very short time.

